# Patient Readiness for Remote Healthcare Services in the Context of the COVID-19 Pandemic: Evidence From European Countries

**DOI:** 10.3389/fpubh.2022.846641

**Published:** 2022-03-15

**Authors:** Marta Borda, Natalia Grishchenko, Patrycja Kowalczyk-Rólczyńska

**Affiliations:** ^1^Department of Insurance, Wroclaw University of Economics and Business, Wroclaw, Poland; ^2^Institute of Social Policy, National Research University Higher School of Economics, Moscow, Russia

**Keywords:** COVID-19 pandemic, digital health, linear ordering methods, healthcare systems, remote healthcare

## Abstract

Despite the fact that remote services were successfully implemented in most European social and health systems before 2020, the COVID-19 pandemic has led to an unprecedented development of health and social care services provided in this form. This paper compares the readiness of patients to use the digital solutions in healthcare systems implemented in EU countries, in response to the current pandemic situation. In the study, technological, health insurance, and demographic variables were selected on the basis of substantive criteria. Next, the linear ordering method was applied to make a ranking of the analyzed countries according to the level of patients' readiness to use digital healthcare services. The main findings show that the Netherlands and Ireland are characterized by the highest level of patients' readiness for using remote healthcare services. On the other hand, Romania and Bulgaria are among the countries with the lowest readiness. The study also made it possible to group European countries according to the level of patients' preparedness.

## Introduction

The COVID-19 pandemic has become a global challenge that has significantly influenced the sustainability of the healthcare systems worldwide. So-called “new normal” causes that health systems have to operate in a challenging context–managing both the increasing demand for healthcare services and the resource constraints with new standards and restrictions (1). In the face of an increasing number of the COVID-19 cases, many countries have introduced strong restrictions on direct access to medical services. Outpatient visits to healthcare providers have been significantly reduced or canceled as they increase the risk of contracting and spreading the virus among healthcare workers, patients and their families (2). Moreover, a decrease in emergency cases in hospitals around the world has been reported, having a significant impact on the health outcomes of the populations (3–5). In the European region, during the pandemic peaks, severe disruptions in delivery of the essential healthcare services have been reported, including among others: Non-communicable disease diagnosis and treatment, rehabilitation services and dental services (1, 6).

Consequently, the implementation of new digital health solutions or development of the existing ones have been observed in most EU countries. For example, in Poland, the results of BioStat CAWI survey indicate that in April 2020 as many as 43.3% of respondents used telemedicine services as a form of medical advice taken in the last 7 days, compared to only 26.7% in March 2020. The interest in all other forms of medical services decreased significantly, and the level of use of emergency medical services remained unchanged (7).

There is no doubt that a positive effect of the ongoing pandemic is the acceleration of digital solutions implementation in EU healthcare systems. Telemedicine and virtual care are considered as an approach to maximize the efficiency of healthcare delivery (8). For the contemporary healthcare systems, it represents one of the most effective potentials to reduce health expenditure, ensure the more effective allocation of resources and improve the access to healthcare services (9). Taking into account the dynamic development of remote healthcare services, the research question arises, if, and to what extent, the patients in the EU are ready to use distance health services as a result of restrictions implemented in healthcare delivery due to the pandemic. The proper assessment of digital health tools is not possible without consideration of the patients' perspective, their readiness, ability and experience to use such services. Consequently, the examination of the patients' preparedness for distance medical services is an important scientific and practical issue.

This paper compares the level of readiness of patients to use the digital solutions in healthcare systems, implemented in selected EU countries, in response to the current pandemic situation. In the study, we formulated the following research hypothesis: There are significant differences among the EU countries, especially between CEE countries and Western European countries, in terms of factors characterizing the patients' ability and experience to use remote healthcare services. The main contribution of the paper is that we made an attempt to evaluate the patients' readiness for remote healthcare services in the context of the COVID-19 pandemic, with the application of linear ordering methods using a Non-model aggregation technique. This approach allows us to create a ranking of the examined countries and indicate the main similarities and differences among them.

## Literature Review

The COVID-19 pandemic has highlighted new challenges in various areas of healthcare services related to their availability, resource mobilization, financing and others. Distance healthcare represents a solution to these challenges due to its ability to provide essential health information and services to vulnerable social groups (elderly, people with disabilities, living in remote areas), monitor morbidity and adhere to social distancing and reduce cases of COVID-19.

In the literature, there are many studies examining various aspects of telemedicine application during the current pandemic, with regard to entire health systems and various areas of medicine, both on the international and regional levels. The research on remote healthcare services is dominated by a systemic approach, in which the potential impact of digital technologies on the healthcare systems in terms of their general goals (including quality, accessibility, efficiency and equity) has been analyzed (10). Remote healthcare services are mainly considered taking into account their cost effectiveness, health outcomes, development prospects in clinical practice, barriers to implementing this type of solutions, and requirements for healthcare providers. A comparative analysis of existing telemedicine frameworks and the factors influencing the implementation of remote medical care in the selected countries worldwide has been presented in (11). In another study the key conditions for effective digital healthcare services implementation in the CEE countries were identified (12). The development of telemedicine (between 1990 and 2020) and the main advantages and disadvantages of remote medical services were examined with regard to the current COVID-19 pandemic and the future (13). Digital technologies in the health systems response to the pandemic, including legal, ethical, financial and privacy barriers to their implementation, have been evaluated in the studies (8, 14, 15). Moreover, the possibilities and the effects of the adoption of telemedicine tools across various medical specialties during and beyond the COVID-19 pandemic are critically examined in (16).

On the other hand, relatively less attention has been paid to the analysis of digital healthcare services from the perspective of patients, their readiness and ability to use such services. In this case, the patient satisfaction surveys are most often carried out in order to evaluate respondents' attitudes toward digital technologies in healthcare delivery. In the research (17), based on data from the New York City, the authors found, with the use of multivariable linear regression, that patient acceptance of video visits compared favorably with in-person visits over the previous year and during the COVID-19 pandemic. Similarly, a cross-sectional study on patient perceptions and satisfaction regarding teleconsultations during the ongoing pandemic in Saudi Arabia indicated that most respondents were satisfied with the use of medical teleconsultations and the main reason for dissatisfaction was the waiting time for a remote consultation (18). A study investigating potential barriers to telemedicine adoption in Bangladesh argues that barriers in organizational effectiveness, health staff motivation, patient satisfaction, and trustworthiness are the most explanatory for the adoption of telemedicine projects (19). A cross-sectional questionnaire-based household survey from Australia on the relationship between e-health access and respondents' characteristics shows that middle age, household size, broadband access and digital literacy increase the likelihood of access, while low educational levels, low socioeconomic status and remote locations negatively affect this access (20). A study on mobile consulting in healthcare in urban and rural settings (Pakistan, Tanzania, Kenya, Nigeria and Bangladesh) finds that the main challenges are in technology, infrastructure, data security, confidentiality, acceptability and health system integration (21). Another study on telehealth adoption in Indonesia shows that performance expectancy, effort expectancy, and facilitating conditions significantly affect behavioral intention to use telehealth, while social influence is not significantly associated with behavioral intention; performance expectancy is also significantly affected by doctor's opinions and effort expectancy is strongly influenced by computer anxiety (22). Authors of a cross-sectional survey in Singapore on tele-monitoring of individuals with type 2 diabetes and/or hypertension, found that 53% of the patients were willing to use telemonitoring (23). This was affected by personal beliefs in technology, prior technology utility, patient's requirements to be accompanied, cost considerations and technological literacy.

Despite the fact that studies of remote healthcare, tele- and e-medicine show important findings and relations, studies on patient readiness for remote health services are lacking on the macro level and with comparison between countries. We suggest that cross-country assessment of patients' readiness for telemedicine services lies in its concept without barriers, universal and accessible with no formal borders between countries, or personal restrictions. In addition, considering that healthcare systems will adapt after the pandemic, it is important to provide an analysis in various areas of assessing patient readiness for remote healthcare, which may include technological factors, demographic characteristics, and the development of the private health insurance sector.

In the paper we take the patients' perspective and examine their readiness to use the digital solutions implemented in the EU healthcare systems, taking into account the selected determinants of patients. There is no doubt that the key group of determinants of the effective use of remote healthcare services are technological factors. Although healthcare providers are ready to offer digital health services to patients, socioeconomic determinants, in particular technological literacy and access, have an essential impact on their effective implementation. The digital domain is becoming the most important due to the widespread use of social services in digital forms. The importance of such technologies in the fight against the COVID-19 pandemic has been highlighted in recent studies: the use of telemedicine and virtual care for remote treatment (8), different approaches for the population surveillance, case identification, contact tracing and the evaluation of interventions on the basis of mobility data and communication with the public (14). There are research-based characteristics of the digital development of the country's population, which include access, skills, and the use of and attitude to digital technologies. Internet access can be defined in different categories as physical or material, in the types of infrastructure, devices, etc. (24). The development of technologies and their adoption by almost all social groups make new technologies more and more accessible. Various digital skills in the use of Internet, devices and applications are essential for remote health care. The importance of digital skills and related behaviors is explored in the context of preventing cyber-victimization (25). It is assumed that for universal distance healthcare, not only digital skills are important, but also digital literacy (26). The use of digital technologies in the context of remote healthcare services means the preference for their use, the frequency of use, and the purpose of using Internet, which may vary depending on the social and economic characteristics of users, but characterize the level of digital penetration for the population of the country quite well (24, 27). The different attitudes of patients toward the use of telecommuting and new technologies should be taken into account (28, 29). As digital healthcare services become more and more universal for all citizens of the country, e-government indicators are important for assessing preparedness. Germany's new Digital Healthcare Act (Digitale-Versorgung-Gesetz or DVG), which was adopted in November 2019, entitles all individuals covered by statutory health insurance to reimbursement for certain digital health applications, i.e., insurers will pay for their use (30). During the COVID-19 pandemic, governments have also explored new ways of using technology (contact-tracing apps, AI chatbots, online permits, E-learning portals) to reach out to and support diverse groups in society (31).

In our study, the technological factors were supplemented with selected additional characteristics of users. We decided to extend the analysis taking into account the objective characteristics, which can be measured with comparable indicators, based on available and verified data. In this approach, the additional factors characterizing patient readiness for remote medical services include: demographic characteristics and the indicators of the level of development of private health insurance (PHI) markets in the examined countries. Subjective factors, such as the tendency to use the Internet, psychological barriers or the level of knowledge about digital healthcare services, were deliberately not included into the analysis.

The demographic factors of respondents affect the effective use of digital technologies, not only in the healthcare sector. In the study evaluating telemedicine unreadiness among older adults in the US, with regards to the demographic variables, such as: age, sex, ethnicity, rurality, marital status, educational level, income and self-rated health status, the authors found that the unreadiness was more prevalent in patients who were older, were men, were not married, were Black or Hispanic, lived in Non-metropolitan areas, with lower educational levels, lower income, and poorer self-reported health (32). In another research, chronic diseases, higher age, lower income, lower educational levels, living alone, and living in rural areas were found to be associated with lower use of digital health tools (33). The importance of demographic characteristics of patients using digital healthcare services is also highlighted, among others, in (9, 34–36).

As mentioned, the indicators characterizing the level of development of the PHI sector in the examined countries were also included into the analysis. The EU countries are differentiated taking into account the size, structure and development of PHI markets, which is mainly determined by the range of the healthcare services provided within the public system. The private health (and life) insurance sector faced the digital transformation before the COVID-19 pandemic. Innovative digital solutions have been implemented into the life and health insurance industry in order to better manage claim processes, fraud detection, policy administration and customer service. So-called “InsurTech” tools can be used in the risk assessment process as diagnostic decision-support, for managing chronic conditions, as well as in everyday healthy life monitoring in line with the “pay as you live” concept (37–40). Consequently, one can expect that people with PHI are more aware of digital tools (such as tele-consultations, digital diagnostic tools, monitoring of vital parameters, e-booking of appointments, prevention tools such as apps, fitnesstracker, etc.) and more experienced in using them compared to the uninsured.

## Methods

To achieve the aim of the paper the linear ordering method, belonging to the methods of multidimensional comparative analysis (41), was applied. The most recent statistical data (for 2018, 2019 or 2020) from Eurostat database, Insurance Europe database and World Bank database for 27 European countries was used.

The basis of the linear ordering method is a synthetic variable, the values of which are estimated on the basis of observations of diagnostic variables describing the examined objects. The synthetic variable allows the comparison and ordering of all objects. Therefore, the first step of the linear ordering procedure is the selection of the diagnostic variables. In this study, the diagnostic variables were selected on the basis of substantive criteria and data availability. Four sub-indicators were created (Table 1). Second, all potential variables were verified in terms of their volatility. Variables characterized by volatility higher than 0.1 were selected. Consequently, three potential diagnostic variables from Internet skills variables group were removed. The final set of diagnostic variables (after the first step of linear ordering) consists of four variables connected with Internet skills, two variables describing private health insurance sector, three demographic variables, and two economic variables.

**Table 1:** Indicators and potential diagnostic variables.

**Name of sub-indicator (Symbol)**	**Symbol of variable**	**Name of variable**	**Value of the variation coefficient**
Internet skills indicator (*ISI*)	*ISI_1_*	Households–level of Internet access as a % of households	0.07
	*ISI_2_*	Individuals' level of digital skills as a % of individuals	0.22
	*ISI_3_*	Individuals' Internet use as a % of individuals	0.08
	*ISI_4_*	Individuals' frequency of Internet use as a % of individuals	0.09
	*ISI_5_*	Individuals who used the Internet for interaction with public authorities as a % of individuals	0.35
	*ISI_6_*	Use of ICT at work and activities performed as a % of individuals	0.27
	*ISI_7_*	Internet purchases by individuals (until 2019) as a % of individuals	0.36
Insurance indicator (*II*)	*II_1_*	PHI penetration ratio (%), total premiums to GDP	2.14
	*II_2_*	Voluntary prepayment as % of current health expenditure	0.96
Demography indicator (*DI*)	*DI_1_*	Proportion of population aged 65 and over (in % of total population)	0.11
	*DI_2_*	At risk of poverty rate (in %)	0.24
	*DI_3_*	Percentage of people with higher education (level 5–8) in the age group 15–74	0.24
Economics indicator (*EI*)	*EI_1_*	Housing cost overburden rate	0.80
	*EI_2_*	Median equivalised net income (in EUR)	0.56

*Own compilation*.

Next, the inverse correlation matrix method was used as a second method of selecting diagnostic variables. Inverse correlation matrixes were created for each group of indicators. While analyzing diagonal elements of inverse correlation matrixes, variables for which the values are lower than 10 should be selected for further calculations. In this study none of the diagnostic variables was removed. The third step of the linear ordering procedure is the stimulation of variables to unify their character, namely to make a higher value more desirable in all variables (42). It means that all variables which are destimulants or nominants have to be changed into stimulants. In this study, only some diagnostic variables were transformed from destimulants into stimulants using the following formula (42):


$$x^{\prime }ij=\underset{i}{\;max}\;x_{ij}-x_{ij}$$

where: *x*_*ij*_ is the value of the *j* variable for the *i* object.

After the stimulation, the normalization of variables was conducted using the unitarization. This led to the unification of variables in the range (0,1).

The unitarization for stimulants was carried out using the following formula (43):


$$\begin{array}{l} z_{ij}=\frac{x_{ij}-\underset{i}{min}{\;x}_{ij}}{\underset{i}{max}{\;x}_{ij}-\underset{i}{min}x_{\;ij}} \end{array}$$

where: *z*_*ij*_ is the unitarized value of the *j* variable for the *i* object; *z*_*ij*_∈〈0, 1 〉.

The last step of the linear ordering procedure was aggregation of sub-indicators, which allowed us to create the synthetic variable (indicator), according to the following formula:


$$\begin{array}{l} SI=\frac{1}{4}\left(ISI+II+DI+EI\right) \end{array}$$

where: *SI* is the synthetic indicator.

*ISI* is Internet skills indicator, which was calculated as arithmetic mean according to the formula:


$$\begin{array}{l} ISI=\frac{1}{4}\left(ISI_{2}+ISI_{5}+ISI_{6}+ISI_{7}\right) \end{array}$$

*II* is Insurance indicator, which was calculated as arithmetic mean according to the formula:


$$\begin{array}{l} II=\frac{1}{2}\left(II_{1}+II_{2}\right) \end{array}$$

*DI* is Demography indicator, which was calculated as arithmetic mean according to the formula:


$$DI=\frac{1}{3}(DI_{1}+DI_{2}+\;DI_{3})$$

*EI* is Economics indicator, which was calculated as arithmetic mean according to the formula:


$$\begin{array}{l} EI=\frac{1}{2}\left(EI_{1}+EI_{2}\right) \end{array}$$

## Results and Discussion

In the study we applied the linear ordering procedure to obtain the values of synthetic indicator characterizing the level of patients' readiness to use remote healthcare services in the particular European countries. In Table 2 the values of synthetic indicator as well the values of sub-indicators are presented.

**Table 2:** Values of sub-indicators and synthetic indicators for EU countries.

**Country**	**Internet skills indicator**	**Insurance indicator**	**Demography indicator**	**Economics indicator**	**Synthetic indicator**
Belgium	0.63	0.21	0.66	0.75	0.56
Bulgaria	0.06	0.04	0.23	0.31	0.16
Czechia	0.53	0.00	0.54	0.56	0.41
Denmark	0.91	0.09	0.64	0.80	0.61
Germany	0.79	0.13	0.42	0.66	0.50
Estonia	0.73	0.00	0.48	0.61	0.45
Ireland	0.55	0.49	0.93	0.84	0.70
Greece	0.35	0.15	0.34	0.05	0.22
Spain	0.52	0.31	0.45	0.58	0.46
France	0.66	0.29	0.59	0.74	0.57
Croatia	0.34	0.15	0.33	0.53	0.34
Italy	0.25	0.08	0.11	0.60	0.26
Cyprus	0.38	0.45	0.79	0.73	0.59
Latvia	0.45	0.05	0.33	0.54	0.34
Lithuania	0.48	0.04	0.49	0.53	0.38
Luxembourg	0.71	0.12	0.76	0.86	0.61
Hungary	0.39	0.07	0.51	0.50	0.37
Malta	0.58	0.08	0.43	0.72	0.45
Netherlands	0.95	0.71	0.63	0.79	0.77
Austria	0.71	0.23	0.61	0.76	0.58
Poland	0.36	0.22	0.54	0.50	0.40
Portugal	0.37	0.25	0.29	0.55	0.36
Romania	0.01	0.04	0.16	0.41	0.16
Slovenia	0.51	0.60	0.59	0.65	0.59
Slovakia	0.49	0.02	0.65	0.53	0.42
Finland	0.83	0.09	0.65	0.83	0.60
Sweden	0.85	0.06	0.59	0.72	0.55

*Own calculation*.

**Figure 1:**
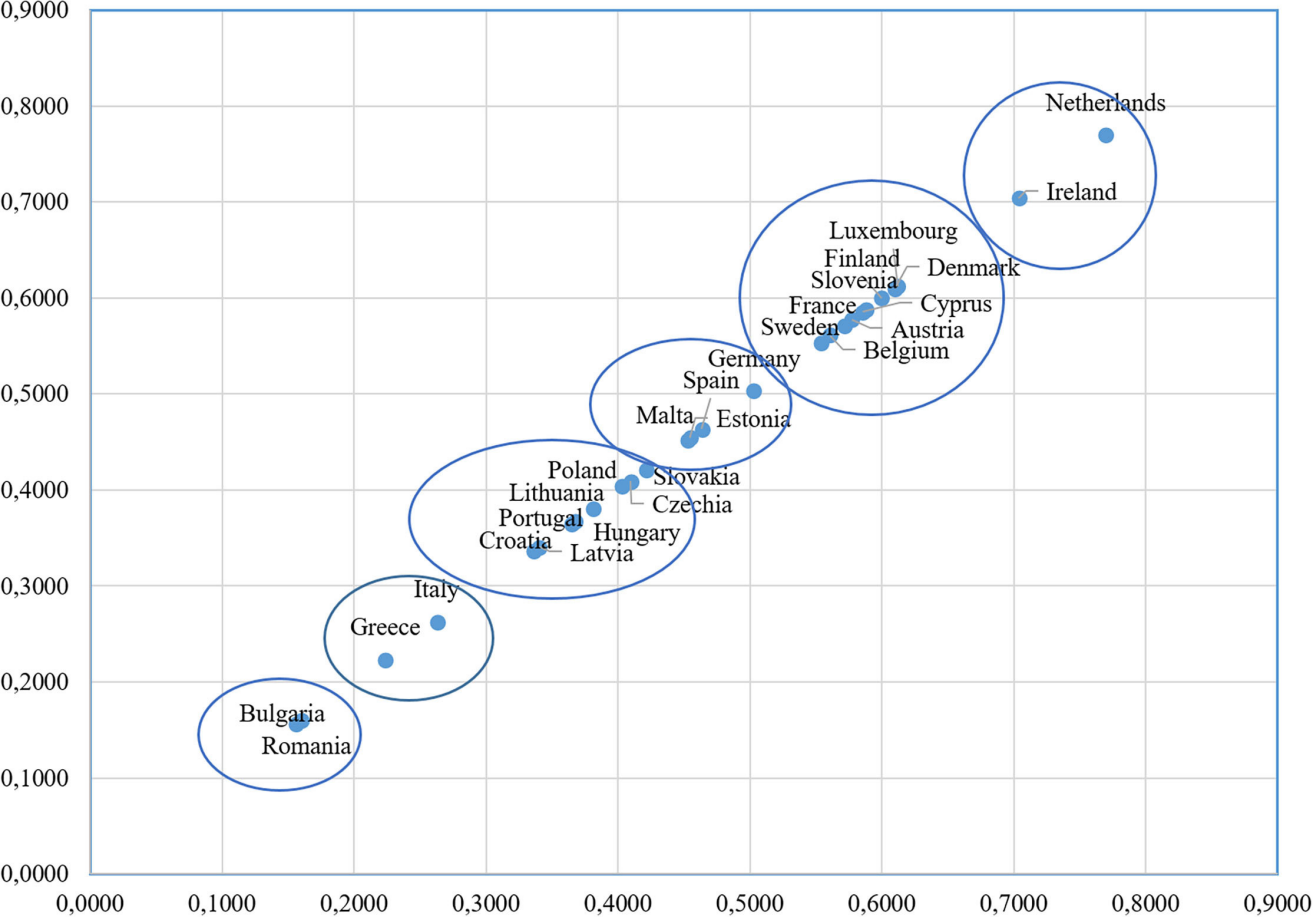
Scatter diagram for a linear ordering of European countries according to the level of patients' readiness for use digital healthcare services. Source: own elaboration.

The obtained values of the synthetic indicator made it possible to order EU countries in terms of the patients' readiness to use remote healthcare services (Figure 1).

The results show that European countries can be divided into six groups. The first group, including Ireland and the Netherlands, is characterized by higher values of median equivalised net income, almost the same values of at risk of poverty rate (~13%), and higher values of Internet purchases by individuals. Luxemburg, Denmark, Finland, Cyprus, Slovenia, Austria, France, Belgium and Sweden belong to the second group. Almost all countries in this group are characterized by similar values of the percentage of people with higher education (level 5–8) in the age group 15–74 (the smallest value is 27.1 % for Slovenia, the highest value equals 36.23 % for Finland). Excluding Luxembourg and Cyprus, all countries in this group are characterized by similar values of proportion of population aged 65 and over (between 18.8 and 21.8%). Excluding Slovenia and Cyprus all countries are characterized by higher values of Internet purchases. We can point out that Cyprus and Slovenia have almost the same values of synthetic indicator. These two countries are characterized by very high values of voluntary prepayment as % of current health expenditure, the similar values of ICT use at work and activities performed, and similar values of individuals who used the Internet for interaction with public authorities. Austria and France are characterized by almost the same values of PHI penetration ratio and risk of poverty rate. Because of that, these two countries have nearly the same value of the synthetic indicator. The third group (Germany, Spain, Malta, and Estonia) is characterized by relatively higher proportions of the population aged 65 and over (18.7–21.5%). Estonia and Spain have similar values of at risk of poverty rate (21.7 and 20.7%, respectively), which are very high compared to other European countries. Estonia and Malta have the same values of ICT use at work and activities performed. The fourth group consists of Poland, Slovakia, Czechia, Lithuania, Latvia, Hungary, Portugal and Croatia. Most of the countries from this group belong to CEE countries. These countries (excluding Portugal) are characterized by relatively low values of PHI penetration ratio. Moreover, in all countries in this group, median equivalised net income values are lower compared with most European countries. As it can be observed Czechia, Hungary and Portugal are characterized by almost the same percentage of people with higher education (level 5–8) in the age group 15–74 (~20.5%). Italy and Greece belong to the fifth group. The proportion of the population aged 65 and over in these two countries is the highest among the considered countries. Values of Internet purchases by individuals are similar for both countries of southern Europe. The last (sixth) group is represented only by Bulgaria and Romania. These two countries are characterized by the lowest values of many indicators, such as individuals' level of digital skills, median equivalised net income, use of ICT at work and activities performed, Internet purchases by individuals. Moreover, risk of poverty rate is the highest for Romania (23.8%) and very high for Bulgaria (22.6%).

As regarding the linear ordering results, among the analyzed countries a significant differentiation in the value of the synthetic indicator is observed. The Netherlands is characterized by the highest value of the synthetic indicator (0.77) and Romania–by the lowest one (only 0.16), respectively. The first five places in the ranking belong to developed Western European countries with relatively effective and well-organized healthcare systems. CEE countries are very similar in the values of the synthetic indicator and they take lowest places in the ranking. In these countries, the availability of many specialized medical services is limited due to organizational and financial barriers in their healthcare systems.

In general, the European countries have used digital health tools in responding to the COVID-19 pandemic as well as they have reorganized non-COVID-19 healthcare service delivery. Some specific initiatives to use digital health tools for remote management of COVID-19 patients with mild symptoms or recuperating at home after hospital care have been implemented in France, Iceland, Italy, Luxembourg, the Netherlands, with also some provision of wider support services for people self-isolating (Poland). Digital tools have also been used to help manage essential health care supplies and hospital bed capacity related to the pandemic (Greece) (44). In the area of non-COVID-19 services, the analyzed countries have either introduced or expanded the use of telemedicine, usually in the form of phone-based consultations (e.g., Croatia, Luxemburg, Romania, Spain), sometimes for specific patient groups (Belgium, the UK). It should be highlighted that an increase in the use of teleconsultations in Belgium and Germany led to the modifications of the benefit basket to allow for more extensive reimbursement of teleconsultations. The use of video-conferencing and other online platforms was also reported. In some EU countries regular prescriptions have been started available by phone (Greece) or e-prescriptions have been introduced or reinforced (Hungary, Ireland, Latvia) [see more in (5)].

On the basis of the conducted analysis and the literature review, it is not possible to indicate a direct relationship between the range of solutions applied by the particular countries in the area of digital healthcare during the first wave of the COVID-19 pandemic and the preparation, possibilities and experience of the patients to use such services. The application of digital healthcare services among the considered European countries is very differentiated in details and depends on the organization of the overall healthcare system rather than the level of patients' readiness for using such services. The obtained results emphasize that, not only the digital characteristics are important, but also demographic and economic factors determine the level of patients' readiness to use remote healthcare services in particular countries. Consequently, in our study the differences between Western European countries and CEE region, in terms of demographic and economic variables influencing the level of patients' readiness, have been confirmed.

## Conclusions

In this article, we make a first attempt to examine patients' readiness for remote healthcare service in the context of the COVID-19 pandemic in Europe. We form the main domains with an evaluation of digital, demographic, PHI and economic predictors which can affect this readiness in the healthcare sector. We find that differences in the patient readiness for remote healthcare service are mainly present between the six groups of countries with high, upper-, middle-, less- and low levels of this readiness.

Along with the state of distance healthcare service, universal for all citizens in the country, the individual readiness of patients for remote medical care is important. However, patient readiness for remote healthcare services varies in multiple way.

We suggest that the readiness of patients for telemedicine services in the indicators under consideration shows a significant differentiation between countries. This may be the cause of inequality in obtaining universal health care in the context of social distancing measures. To a certain extent, we can conclude that the pandemic exacerbates the situation with the availability of medicine due to different levels of patient readiness for remote healthcare. Public policy should include activities to improve the availability of telemedicine services in critical medical situations. Policymakers should monitor patient readiness for remote health services and take the necessary steps to bridge inequalities in this area.

As regards the potential future research, we suppose that the refinement and expansion of the set of determinants for assessing patients' readiness for remote healthcare will lead to more accurate findings. We suggest taking into account such a factor as the size of the country, which is important for assessing remoteness when studying remote health services. It will also be important to compare the readiness of patients for remote medical care with the number of cases of the COVID-19 pandemic by country in different periods of 2020–2022.

## Limitations

Since the purpose of this article is to assess the readiness of patients for remote health services in a medical emergency, we used only some of the related determinants of such readiness in a specific situation. Other limitations are generally related to the methodologies for selecting potential indicators and their availability. We use macro level indicators, which can also limit the estimates based on the study's results.

## Data Availability Statement

The original contributions presented in the study are included in the article/Supplementary Material, further inquiries can be directed to the corresponding author.

## Author Contributions

MB, NG, and PK-R: conceptualization and writing. MB and NG: literature review. PK-R: analysis. MB: editing. All authors contributed to the article and approved the submitted version.

## Funding

This project is financed by the Ministry of Science and Higher Education in Poland under the programme Regional Initiative of Excellence 2019–2022 project number 015/RID/2018/19 total funding amount 10 721 040,00 PLN.

## Conflict of Interest

The authors declare that the research was conducted in the absence of any commercial or financial relationships that could be construed as a potential conflict of interest. The handling editor declared a past collaboration with one of the authors MB.

## Publisher's Note

All claims expressed in this article are solely those of the authors and do not necessarily represent those of their affiliated organizations, or those of the publisher, the editors and the reviewers. Any product that may be evaluated in this article, or claim that may be made by its manufacturer, is not guaranteed or endorsed by the publisher.
